# Assessing Diversity and Inclusivity is the Next Frontier in Mental Health Recovery Narrative Research and Practice

**DOI:** 10.2196/44601

**Published:** 2023-04-17

**Authors:** Yasuhiro Kotera, Stefan Rennick-Egglestone, Fiona Ng, Joy Llewellyn-Beardsley, Yasmin Ali, Chris Newby, Caroline Fox, Emily Slade, Simon Bradstreet, Julian Harrison, Donna Franklin, Olamide Todowede, Mike Slade

**Affiliations:** 1 School of Health Sciences Institute of Mental Health University of Nottingham Nottingham United Kingdom; 2 School of Medicine Queen’s Medical Centre University of Nottingham Nottingham United Kingdom; 3 Department of Computer Science University of Oxford Oxford United Kingdom; 4 School of Health & Wellbeing University of Glasgow Glasgow United Kingdom; 5 Narrative Experiences Online Lived Experience Advisory Panel Nottingham United Kingdom; 6 Faculty of Nursing and Health Sciences Health and Community Participation Division Nord University Namsos Norway

**Keywords:** recovery narrative, web-based mental health interventions, inclusivity, diversity, collective action, curation, mental health, digital health, telemedicine, clinical practice, narrative research, demographic

## Abstract

Demand for digital health interventions is increasing in many countries. The use of recorded mental health recovery narratives in digital health interventions is becoming more widespread in clinical practice. Mental health recovery narratives are first-person lived experience accounts of recovery from mental health problems, including struggles and successes over time. Helpful impacts of recorded mental health recovery narratives include connectedness with the narrative and validation of experiences. Possible harms include feeling disconnected and excluded from others. Diverse narrative collections from many types of narrators and describing multiple ways to recover are important to maximize the opportunity for service users to benefit through connection and to minimize the likelihood of harm. Mental health clinicians need to know whether narrative collections are sufficiently diverse to recommend to service users. However, no method exists for assessing the diversity and inclusivity of existing or new narrative collections. We argue that assessing diversity and inclusivity is the next frontier in mental health recovery narrative research and practice. This is important, but methodologically and ethically complex. In this viewpoint, we propose and evaluate one diversity and two inclusivity assessment methods. The diversity assessment method involves use of the Simpson Diversity Index. The two inclusivity assessment methods are based on comparator demographic rates and arbitrary thresholds, respectively. These methods were applied to four narrative collections as a case study. Refinements are needed regarding a narrative assessment tool in terms of its practicality and cultural adaptation.

## Introduction

### Background

Demand for digital mental health interventions (DMHIs) has been increasing in many countries [1]. For example, 76% of the Australian general public indicated an interest in using DMHIs [2], and US $2.4 billion were invested in these interventions in 2020 alone in the United States [3]. A Singaporean mental health app, Intellect, is now used by over 3 million people in 20 countries, mostly in Asia [4]. A large-scale education program about ethics in DMHIs has been launched [5], and more active use of DMHIs is expected in Africa [6]. The advantages of DMHIs include accessibility, cost-effectiveness, and personalization [7], which can address key barriers for mental health recovery, such as low help-seeking and the stigma associated with mental health problems [8]. Effectiveness of DMHIs has been generally reported in diverse populations (eg, children, young people, older adults, university students, health care workers, people with neurodevelopmental disabilities) and on diverse mental health experiences [2,9-12]. DMHIs have become an important domain in medical internet research [13-15].

Mental health recorded recovery narratives (RRNs) have been used in DMHIs. Mental health recovery narratives can be defined as “first-person lived experience accounts of recovery from mental health problems, which refer to events or actions over a period of time, and which include elements of both adversity/struggle and of self-defined strengths/successes/survival” [16]. Although narratives can be shared in person, such as when a peer support worker tells their story [17], RRNs are those presented in recorded formats such as written text, audio, and video. RRNs are often disseminated online [18] and have been used in a range of clinical and societal interventions [19]. For example, the Narrative Story Bank was created by the Scottish Recovery Network to inspire hope and offer tools and technologies for recovery [20]. Content from the Narrative Story Bank helped to inform Scotland’s mental health strategy [21]. In Scotland, the use of recovery narratives has been incorporated into clinical practice, such as the production of written RRNs to support self-reflection [21]. Similarly, RRNs have been used in clinical training to enhance communication skills and empathy of health care workers [22]. RRNs are used in national antistigma campaigns [23], which aim to reduce stigma associated with mental health problems [24].

The impact of RRNs on recipients has been evaluated. For example, students who listened to recovery narratives of people with anorexia nervosa showed more understanding of the mental illness and a less stigmatized view toward these individuals [25]. Eating disorder memoirs offered validation of personal experience and created a positive affective response among people with eating difficulties [26]. Helpful outcomes of accessing both live and recorded recovery narratives include connection with others, validation of own experiences, empowerment, hopefulness, gratitude, and stigma reduction [27]. Mechanisms of impact from RRNs include comparison with the narrative and narrator, learning about the experiences of others, and feeling empathy, all of which create a sense of connection [28]. Accessing RRNs can thus support personal recovery by increasing hope, meaning, and a sense of fulfillment [18,29].

RRNs are often grouped and presented as collections [19], which have been provided on bespoke websites [30] and in books composed of recovery narratives linked by a theme such as psychosis [31] or men’s eating disorders [32]. The people who assemble RRN collections do so for a range of reasons, including providing education about mental health and recovery, supporting others in their recovery journey, and campaigning for change in health service provision [33].

Clinicians who use RRNs as part of treatment need to know the possible impact of the RRN collections they recommend to their service users [34]. Given their broad range of clinical applications, the ability to characterize a narrative collection is important because not all RRNs are helpful to all people. An interview study reported that recovery narratives can be harmful if recipients feel disconnected from the narrative, resulting in distancing themselves from the narrator [27]. For instance, if a “right” way to recover is communicated by the RRNs, this implicit message can cause a recipient to feel disconnected [35,36] if that particular pathway is in some way “outside” that person’s scope, experience, and perspective. A feeling of being disconnected is detrimental because connection is the central mechanism supporting recovery after access to RRNs [28,37].

### Diversity and Inclusivity

Providing diversity in RRN collections may be one approach to maximizing benefit and minimizing harms, enabling a sense of connection to people with different backgrounds and experiences. A diverse set of narratives will increase the opportunity for the recipient to feel connected to a narrative [27]. Further, a lack of diversity in RRN collections may give rise to stereotyping [38], leaving recipients feeling excluded and disconnected [18], or suggest that there is only one way or a “right” way to recover [35,39], which may not fit with their own beliefs, experiences, and needs. Recipient characteristics and narrative characteristics moderate the impact of a narrative on a sense of connection [28]. For example, people in ethnic minority groups felt a lower level of connection when an RRN collection marginalized these groups [40]. Diverse narratives are needed to ensure that recipients from different backgrounds and with different identities have the greatest opportunity to feel connected with both a narrative and a narrator, as both types of connection are known to maximize the positive effects of RRNs [38]. One exception might be collections of RRNs targeted at specific groups experiencing structural inequalities or marginalization (eg, refugees), where the selection of narrators who belong to those groups might be an appropriate strategy for maximizing beneficial impact.

Two types of variation in a narrative collection can be differentiated: diversity and inclusivity [41]. *Diversity* is defined as heterogeneity in narrative characteristics, such as a spread of narrator demographics and “protected characteristic” identities, as defined by the Equality Act 2010 (eg, age, gender, sexual orientation), or types of narrative content (eg, trajectory, genre). *Inclusivity* is defined as representativeness of narrative characteristics in relation to a broader population [42].

Diversity and inclusivity concepts can be applied to a specific characteristic of an RRN collection, such as narrator age or a narrative focused on a certain demographic characteristic (eg, race, disability). Diversity is present when there are a meaningful number of narratives within each subcategory [43]. For example, diversity in narrator age is present when there is a spread of younger, middle-aged, and older narrators. Since diversity is a feature of the collection, a diversity metric will be a constant for a given collection. By contrast, inclusivity is present when the proportion of narratives within each subcategory is similar to the proportion in a wider comparison population at a given time [44]. For example, inclusivity in narrator age is present when there are similar proportions of each narrator age group in the collection and in a comparison population, such as other people on the caseload of the mental health service or in the general population. Since inclusivity is a function of the collection when used in a specific context, an inclusivity metric will vary based on context.

We argue that assessing diversity and inclusivity of narrative collections is the next frontier in mental health recovery narrative research and practice. A tool to characterize individual RRNs has been developed. The Inventory of Characteristics of Recovery Stories (INCRESE) is a standardized 77-item instrument characterizing narrative mode, narrator and narrative characteristics, content warnings, turning points, and narrative content [45]. Although INCRESE is used to characterize individual RRNs, no method currently exists to assess the diversity and inclusivity of an RRN collection.

We here discuss aspects of measuring diversity and inclusivity in recovery narrative collections. The Narrative Experiences Online (NEON) study used INCRESE to characterize a large collection of 687 recovery narratives. Because we had a large data set of narrative characteristics measured using INCRESE available, we used this data set for the present analysis.

### Ethics Approval

The NEON study received approval from a UK National Health Service Research Ethics Committee (West London and GTAC, 18/LO/0991).

## Identifying the Relevant Characteristics

To develop diversity and inclusivity metrics, characteristics for the evaluation of diversity and inclusivity need to be identified. To establish a theoretical and cross-culturally valid understanding of important diversity and inclusivity characteristics, we analyzed policy and research to identify characteristics that are internationally agreed as requiring protection from discrimination. Three data sources were used. First, national policy documents relating to equality, diversity, and inclusivity were reviewed to identify characteristics protected by law in each country. Policy documents were collated from a purposive sample of 20 predefined countries shown in Table 1, chosen for variation in (1) region; (2) income level as classified by the World Bank; and (3) status as a Western, Educated, Individualized, Rich and Democratic (WEIRD) versus non-WEIRD country [46] (see Multimedia Appendix 1 for income levels and WEIRD/non-WEIRD status). Policy documents were retrieved using Google searches with the terms “antidiscrimination [country]” and “human rights [country].” At least one source of information was identified for each country. Where the governmental information was not available in English (eg, Iran, Yemen, Morocco), online sources such as information websites (eg, The Academic Network of European Disability Experts, Human Rights Watch, European Commission, International Labor Organization) or reports (eg, Human Rights Committee Report, Human Rights Watch Report) written in English about protected characteristics in the country were reviewed. The identified characteristics were grouped, and Table 1 shows the frequency across the 20 countries of the 13 identified characteristics protected by law and policy.

Some characteristics collapse complex and contested components, where terms are used inconsistent internationally. For example, “Sex and gender” refers to both biological sex assigned at birth and the social construct gender, with subcategories including “female,” “male,” and “nonbinary” [47]. Therefore, the theme is categorized as “Sex and gender” [48].

The five characteristics with the highest international consensus are Sex and gender (eg, assigned sex at birth, socially constructed gender, female/male/nonbinary), Beliefs (eg, political, religious, philosophical), Origin (eg, race, ethnicity), Family (eg, marital status, carer responsibilities), and Disability (mental, physical, learning, and sensory).

To maximize cross-cultural validity, four multinational documents were reviewed, comprising two international human right treaties (Universal Declaration of Human Rights, Convention on the Rights of Persons with Disabilities) and two relevant systematic reviews about diversity and inclusivity [49,50]. The presence of each of the 13 identified characteristics in these four documents was tabulated to identify the most widely agreed characteristics relevant to diversity and inclusivity (Table 2). In both tables, the authors YK and FN independently reviewed the documents and discussed the rating until consensus was reached, which was then confirmed by the other authors.

**Table 1:** Characteristics (N=13) protected by law and policy in 20 countries.

Country	Sex and gender	Beliefs^a^	Origin^b^	Family^c^	Disability	Sexuality	Age	Economics^d^	Employment	Pregnancy	Education	Language	Military veteran
Australia	✓		✓		✓	✓	✓		✓				
Brazil	✓	✓	✓	✓	✓	✓	✓		✓	✓			
Cuba	✓	✓											
Greece	✓	✓	✓	✓					✓				
Guyana	✓	✓	✓	✓	✓		✓	✓	✓	✓			
Iran	✓	✓	✓			✓						✓	
Ireland	✓	✓	✓	✓	✓	✓	✓	✓					
Italy	✓	✓	✓					✓				✓	
Japan	✓	✓	✓	✓				✓			✓		
Libya	✓	✓	✓										
Morocco	✓	✓		✓									
Netherlands	✓	✓	✓	✓	✓	✓	✓	✓	✓				
Norway	✓	✓	✓	✓	✓	✓	✓			✓			
Palestine	✓	✓									✓		
Spain	✓	✓	✓		✓								
Suriname	✓	✓	✓					✓			✓	✓	
Tunisia	✓		✓		✓			✓					
United Kingdom	✓	✓	✓	✓	✓	✓	✓			✓			
United States	✓	✓	✓	✓	✓	✓	✓			✓			✓
Yemen	✓	✓	✓	✓				✓					
Count	20	18	17	11	10	8	8	8	5	5	3	3	1

^a^Includes religious, political, and philosophical beliefs.

^b^Includes race, ethnicity, migration.

^c^Includes marriage status and carer responsibilities.

^d^Includes social class.

**Table 2:** Candidate diversity and inclusivity characteristics mapped against international treaties and systematic reviews.

Characteristic	UDHR^a^ article	CRPD^b^ article	Yadav and Lenka [49]	Manoharan and Singal [50]
Sex and gender	✓	✓		✓
Beliefs	✓	✓		
Origin	✓		✓	✓
Family	✓	✓		✓
Disability		✓		
Sexuality			✓	
Age		✓	✓	✓
Economics	✓	✓		
Employment	✓	✓		
Pregnancy				
Education	✓	✓		
Language	✓			✓
Military veteran				

^a^UDHR: Universal Declaration of Human Rights.

^b^CRPD: Convention on the Rights of Persons with Disabilities.

All characteristics apart from “Pregnancy” were identified by at least one of the four sources. Finally, to maximize relevance to mental health recovery narratives, a systematic review making recommendations for best practice in curating mental health lived experience narrative collections was assessed [38]. “Positioning” was added as a mental health narrative–specific characteristic to assess if a collection includes both positive and negative narratives about mental health services to capture whether a broad range of perspectives are included [38]. In total, these 14 characteristics were identified as relevant to the diversity and inclusivity of RRN collections.

## Mapping Against a Narrative Characterization Tool

To enable an assessment of the appropriateness of INCRESE in assessing diversity and inclusivity, the 77 INCRESE items were mapped against the 14 diversity and inclusivity characteristics (Table 3).

Twenty-two INCRESE items were able to be mapped against the diversity and inclusivity characteristics. No INCRESE items were identified relevant to the characteristics of Language and Military veteran. Twelve characteristics, including all five of the most supported characteristics and Positioning, can be measured using the INCRESE items. Our INCRESE database enabled a preliminary investigation of diversity and inclusivity, despite INCRESE not being a perfect tool for assessing these metrics. The approach may be refined in the future by including items regarding language and military status.

**Table 3:** Inventory of Characteristics of Recovery Stories (INCRESE) items mapped against the diversity and inclusivity characteristics.

Characteristic	Corresponding INCRESE items
Sex and gender	11 Gender
Beliefs	71 Activism, 72 Spiritual/religious activities
Origin	13 Ethnicity, 15 Location
Family	49 Family, 53 Relationships, 74 Caring responsibilities, 75 Family experiences of mental health issues
Disability	17 Visual difficulties, 18 Hearing difficulties, 19 Mobility/stamina difficulties, 20 Cognitive difficulties, 21 Self-care difficulties
Sexuality	16 Sexuality
Age	12 Age
Economics	54 Income, 55 Housing
Employment	56 Work
Pregnancy	48 Pregnancy/birth
Education	51 Education
Language	Not applicable
Military veteran	Not applicable
Positioning	32 Positioning

## Quantifying Each Characteristic

To quantify the diversity and inclusivity of RRN collections, each characteristic needs to be assessed using its subcategories (eg, for the Sex and gender characteristic, the INCRESE characterization choices of “Male,” “Female,” and “Other” may be the subcategories). Measuring diversity involves characterizing the spread of narratives across each subcategory. For example, an RRN collection that includes no narrator categorized as “Other” in the Sex and gender characteristic is less diverse than an RRN collection that does include such narrators. By contrast, measuring inclusivity involves establishing the same two parameters of characteristics and subcategories, and additionally identifying the comparison population. For example, presence of an Origin subcategory of “white” may not increase an inclusivity metric in the UK general population as much as it does in many other populations.

## Measurement of Diversity and Inclusivity

### Overview

We present one option for measuring diversity and two options for measuring inclusivity for recovery narrative collections. The two options for measuring inclusivity have different properties, enabling people assessing inclusivity to make a choice over which to use.

### Measuring Diversity: Simpson Diversity Index

The Simpson Diversity Index (SDI) is an established index used in the natural sciences to assess biodiversity [43]. The SDI considers the number of species present and the abundance in each species to indicate the variance in species. The SDI is calculated by deducting the Simpson Index (SI) from 1, where SI=Σ*n*(*n*–1)/*N*(*N*–1). When used to assess narrative collection diversity in relation to a particular characteristic (eg, “narrator gender”), *n* refers to the total number of narratives within each option of the characteristic (eg, “female narrator”) and *N* refers to the total number of narratives across all options. See Multimedia Appendix 2 for example calculations. The SDI ranges from 0 (low diversity) to 1 (high diversity).

### Measuring Inclusivity Option 1: Demographic Rates as Comparison Population

Inclusivity captures the extent to which minority groups in a comparison population (eg, a country population, a service user cohort at one mental health service) are included in a collection [51]. One approach to assess inclusivity is to identify the categories that are minoritized in the comparison population and compare their proportion in the narrative collection. For example, the Origin characteristic is measured by INCRESE item 13 “Ethnicity,” with categories of “Not identifiable,” “Asian,” “Black/African/Caribbean,” “Dual/multiple ethnic group,” “Other ethnic group,” and “white.” When used in the United Kingdom, all choices apart from “white” are minority groups (a limitation of this categorization is that some “white” communities such as the Traveler, Gypsy, and Roma communities are also very socially excluded). In the United Kingdom, 13% of the population are nonwhite [52]. In a collection, among all narratives, if the ratio of nonwhite narratives is higher than 13%, the collection can be considered as inclusive with respect to ethnicity in the United Kingdom. If inclusivity is being assessed in a different comparison population such as a different country, then alternative choices for minority categories would be made.

### Measuring Inclusivity Option 2: Arbitrary Threshold as Benchmark

A second approach to measuring inclusivity is to set an arbitrary benchmark. One approach is to decide that five narratives are sufficient to satisfy a benchmark for a certain characteristic. A stronger approach, as often used for external examination in the university sector [53,54], is to decide both a minimum rate (eg, 10%) and number (eg, 5), and choose whichever is greater.

## Case Study

### Data Set, Analysis, and Outcomes

The three methods above were applied to the NEON Collection as a case study. Four characteristics matching INCRESE items were considered: Sex and gender, Origin, Disability, and Positioning. The NEON Collection is a curated collection of mental health RRNs. All narratives are included in the NEON Collection with permission [55]. Each narrative is characterized using INCRESE by multiple raters [45].

The candidate approaches were applied to four groups: the entire NEON Collection, two of the larger collections chosen for difference in source, and the individual donations contained in the NEON Collection. In September 2022, the NEON Collection comprised 687 narratives compiled from 34 public collections and from individual donations. One of the two larger collections, which we here refer to as “statutory service” (78 narratives), was compiled by a statutory mental health service. The other, which we refer to as “ethnic minority book” (19 narratives), was published as a book focusing on the mental health of ethnic minority groups. Individual donations (n=29) comprised narratives collected directly from individuals as donations to the NEON Collection.

To evaluate diversity using the SDI, because the Origin and Disability characteristics consist of multiple INCRESE items, the mean SDI scores were calculated. The SDI scores for each collection group are shown in Table 4.

**Table 4:** Diversity scores (Simpson Diversity Index) for Sex and gender, Positioning, Origin, and Disability characteristics.

Characteristics and corresponding INCRESE^a^ items		NEON^b^ collection	Statutory service	Ethnic minority book	Donations
Sex and gender (INCRESE=Gender)		0.58	0.60	0.29	0.64
Positioning		0.61	0.56	0.56	0.53
**Origin**					
	Location	0.70	0.50	0.20	0.57
	Ethnicity	0.58	0.07	0.61	0.46
	Mean^c^	0.64	0.37	0.51	0.52
**Disability**					
	Visual	0.01	0.00	0.00	0.00
	Hearing	0.01	0.00	0.00	0.00
	Mobility	0.01	0.03	0.00	0.00
	Cognitive	0.01	0.07	0.00	0.07
	Self-care	0.05	0.05	0.11	0.00
	Mean^c^	0.04	0.03	0.02	0.01

^a^INCREASE: Inventory of Characteristics of Recovery Stories.

^b^NEON: Narrative Experiences Online.

^c^Multiple INCRESE items are attached to one characteristic.

Of the four groups assessed, the NEON Collection is the most diverse in terms of Positioning, Origin, and Disability. Individual donations are the most diverse in terms of Sex and gender. Statutory service is the most diverse in the Disability subcategory of cognitive difficulties. Ethnic minority book is the most diverse in the Original subcategory ethnicity.

To evaluate inclusivity using option 1 (demographic rates), the comparison population used was the UK general population. The Positioning characteristic was excluded as there are no demographic data of narrative positioning available. Minority groups in each characteristic were identified and the proportion of narratives from each minority group was calculated. The proportions of minority groups in the comparison UK general population were obtained for gender [56], ethnicity [52], and each Disability component [57-61]. For location, the number of British nationals living outside Europe was identified and then the proportion against the UK population was calculated [62]. For self-care, the prevalence of self-neglect was identified [61]. The findings are shown in Table 5.

**Table 5:** Inclusivity option 1: minority group proportions compared to the UK general population.

Characteristic and INCRESE^a^ item			Response categories				Proportion of minority narratives (%)									Minority proportion in UK general population (%)
			Nonminority		Minority		NEON^b^ Collection		Statutory service		Ethnic minority book		Donations			
Sex and gender (INCREASE=Gender)			Not identifiable, Female, Male		Other		1		0		0		0		3	
**Origin**																
	Ethnicity	Not identifiable, white		Asian, Black/African/Caribbean, Dual/multiple ethnic group, Other ethnic group		10		0		*47* ^c^		0		13		
	Location	Europe		Africa, Asia, Australasia, North America, South America		*36*		0		3		3		7		
**Disability**																
	Visual difficulties	Not identified		Yes		0.4		0		0		0		3		
	Hearing difficulties	Not identified		Yes		0.3		0		0		0		17		
	Mobility	Not identified		Yes		3		1		0		0		46		
	Cognitive difficulties	Not identified		Yes		*4*		*4*		0		*3*		2		
	Self-care	Not identified		Yes		*2*		*3*		*5*		0		0.2		

^a^INCRESE: Inventory of Characteristics of Recovery Stories.

^b^NEON: Narrative Experiences Online.

^c^Numbers in italics indicate proportions above those of the UK general population.

The NEON Collection met the inclusivity benchmark of being above the UK general population for the location subcategory in the Origin characteristic and for two subcategories in the Disability characteristic: cognitive difficulties and self-care. Likewise, statutory service met the inclusivity benchmarks for cognitive difficulties and self-care. Ethnic minority book met the inclusivity benchmarks for ethnicity in the Origin characteristic and self-care in the Disability characteristic. Individual donations met the inclusivity benchmark for cognitive difficulties. No collections met the inclusivity benchmarks for the Sex and gender characteristic or the Disability subcategories of visual difficulties, hearing difficulties, and mobility. The largest inclusivity score was for ethnicity in the ethnic minority book, followed by location in the NEON Collection.

To evaluate inclusivity using option 2 (arbitrary threshold), benchmarks were set at a minimum proportion of 10% of the number of narratives in a collection and a minimum number of 5 narratives. Whichever was the higher number was used as the threshold, as shown in Table 6.

The NEON Collection met the inclusivity benchmarks for both ethnicity and location in the Origin characteristic and for the Positioning characteristic. Ethnic minority book met the inclusivity benchmark for ethnicity in the Origin characteristic. Neither statutory service nor individual donations met any of the characteristics or subcategories. Similar to inclusivity option 1, the scores for location in the NEON Collection and ethnicity in the ethnic minority book markedly exceeded the benchmarks.

**Table 6:** Inclusivity option 2: arbitrary thresholds.

Characteristics and corresponding INCRESE^a^ items			NEON^b^ Collection (n=687); threshold: n=69		Statutory service (n=78); threshold: n=8		Ethnic minority book (n=19); threshold: n=5		Individual donations (n=29); threshold: n=5
Sex and gender (INCRESE=Gender)			4		0		0		0
**Origin**									
	Ethnicity	*69* ^c^		0		*9*		1	
	Location	*245*		0		0		1	
**Disability**									
	Visual	3		0		0		0	
	Hearing	2		0		0		0	
	Mobility	18		1		0		0	
	Cognitive	26		3		0		1	
	Self-care	17		2		1		0	
Positioning			*97*		5		4		2

^a^INCRESE: Inventory of Characteristics of Recovery Stories.

^b^NEON: Narrative Experiences Online.

^c^Numbers in italics indicate proportions above the threshold.

### Strengths and Limitations of Each Approach

#### Simpson Diversity Index

A strength of calculating the diversity scores using the SDI is its practicality; to calculate the SDI, only the frequencies for each characteristic are required [63]. Less practical measures exist. For example, the Shannon Diversity Index is another established biodiversity index, which requires more data such as the rate of each species present out of the total organism population [64]. Likewise, Social Choice Methods were proposed in computer science; however, these methods require more data than required for the SDI (eg, social structures of power and influence) [65].

Three major weaknesses need to be noted. One is that there are no interpretation scores to indicate a level of the diversity in the SDI (eg, high, medium, and low). Another limitation is reliance on INCRESE data, which do not map onto all protected characteristics identified. In particular, there are no INCRESE items for Language and Military status. Moreover, even where an INCRESE item and a characteristic have the same label, the meaning may be different. For example, in this analysis, we chose an INCRESE item for location as part of the Origin characteristic. However, the response choices of this item are placed at a global level (eg, Europe, Asia, Africa), whereas the international treaties and national policies often regard the location of where people are from or live at a local level (eg, housing discrimination in the United Kingdom [66] and “buraku” [roughly defined as a defiled area] in Japan [67]). Both items and response choices in INCRESE can be extended to enable calculation of the diversity from the INCRESE data set. Lastly, while appealing as a simple and comprehensible metric, the SDI may misleadingly simplify the complex issue of diversity. For example, the diversity scores should not be treated as a target, which can be deprioritized once hit [68]. The diversity scores should rather be used as part of helping recipients from different backgrounds and with different identities feel connected with both a narrative and a narrator, maximizing the positive effects of RRNs [38].

#### Inclusivity Option 1

A strength of inclusivity option 1 is that it is a logical approach, comparing the proportion between a collection and its comparable population. This method allows a direct comparison with different contexts as far as the demographic data are available, and tailors the assignment of minoritized status to categories matching the comparison population.

Weaknesses include the time required, modest collection sizes, and comparator choice. First, inclusivity option 1 requires more time than option 2, because of the difficulties with finding the comparable data. There would be a health service resource allocation implication of choosing option 1 rather than option 2. Busy practitioners may not have time to identify comparable demographic data. For example, for self-care, finding the demographic proportion of people with self-care difficulties required a great amount of time. We used the demographic proportion of people suffering from self-neglect. Self-care difficulties and self-neglect may be similar; however, self-neglect can indicate a wider set of behaviors than self-care, such as hoarding and unwillingness to receive support [69,70]. Second, RRN collections often do not have many narratives (eg, the largest collection in the NEON Collection includes 78 narratives). Only a few narratives can meet the benchmarks in small collections. For example, in the ethnic minority book (n=19), only one narrative is sufficient to meet the benchmarks for the subcategories of gender, and visual, cognitive, and self-care difficulties, despite each having different demographic proportions. Lastly, a decision needs to be made on what an appropriate comparator is for this method. We used the UK general population; however, if a statutory mental health service uses this method, the entire cohort of their service users may be more meaningful as a comparative tool. Identifying a meaningful comparator and retrieving comparison information may be complex [71].

#### Inclusivity Option 2

Strengths of inclusivity option 2, based on the arbitrary threshold, include practicality and representation. This approach allows a reasonable minimum number to be present, addressing the need for individuals from minority communities to “see themselves” [72] in the narrative collection.

However, weaknesses include the difficulty in justifying the benchmark numbers. Relatedly, the approach produces a binary outcome: the collection is either inclusive in a particular characteristic or it is not. This does not differentiate between a collection that just meets the benchmark versus one that markedly exceeds it.

Overall, the diversity and inclusivity of the NEON Collection are higher than those of the three subgroups. One explanatory attribute is its size, as the three subgroups are part of the NEON Collection. Because the size is large, the NEON Collection has an inherent advantage with respect to diversity [73]. The more narratives a collection has, the more likely the collection will have different types of narratives. The size advantage also relates to the high inclusivity of the NEON Collection. The NEON Collection marked low yet above-benchmark scores in the characteristics where the benchmarks were low, whereas the other three collections did not (eg, scored a 0 indicating no relevant narrative identified).

Our case study has three implications. First, the diversity and inclusivity can be measured, although each metric has limits on its meaningfulness. For example, the diversity metric has no interpretation scores to indicate a level of the diversity. Inclusivity option 1 highlights a mismatch between INCRESE items and characteristics, and raises the question about defining an appropriate comparator. Inclusivity option 2 requires justification for the benchmark. Second, larger collections in general are more diverse and inclusive. Third, collections that have a specific focus (eg, on ethnicity) can be differentiated using these metrics.

**Figure 1:**
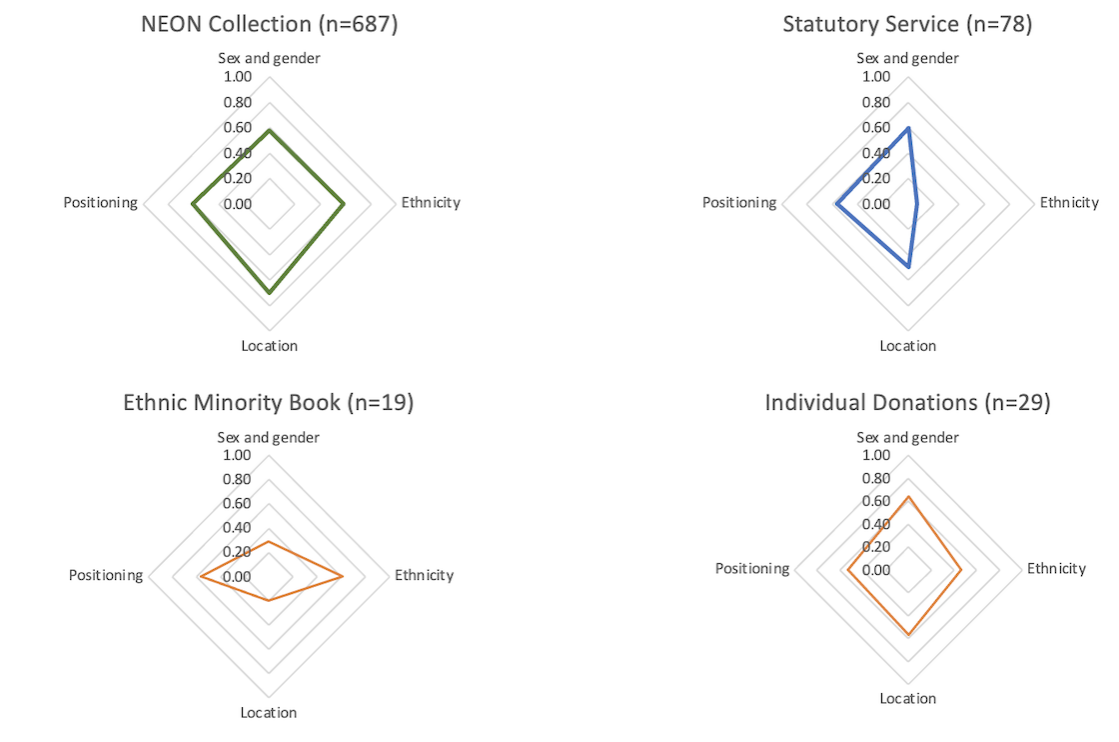
Radar charts to present the diversity scores for the Sex and gender, Origin (Ethnicity and Location), and Positioning characteristics in four collections. The radar chart presentation can visually highlight the uniqueness of each collection, and can be more reader-friendly and inclusive, but cannot present many characteristics. NEON: Narrative Experiences Online.

Finally, how the output is presented needs to be discussed. A challenge with all measurement approaches is how to present the results [74]. One approach is a radar chart, used in the Four Layers of Diversity Model [75]. For example, the Sex and gender, Origin (ethnicity and location), and Positioning characteristics can be presented as shown in Figure 1. The items in the Disability characteristic were excluded as all five items included a 0, which is already visible in the table format.

One advantage of the radar chart is that the uniqueness of each collection can be visually highlighted [76]. Moreover, compared to a table format, chart formats such as a radar chart are often more reader-friendly and inclusive (eg, for people with dyslexia) [77,78]. A disadvantage is that not many characteristics can be included to maintain a reader-friendly presentation [76].

## Conclusion

Several knowledge gaps exist. First, there is a mismatch between the INCRESE items and the identified characteristics. Candidate new items for INCRESE are Language and Military veteran. Moreover, there are currently only three characterization choices for Sex and gender in INCRESE (“male,” “female,” and “other”). More diverse and inclusive choices are needed (eg, “transgender,” “nonbinary/nonconforming”). The content of INCRESE might be reviewed to enhance its use in diversity and inclusivity metrics. Second, the assessment of inclusivity requires development of a reliable and context-sensitive approach to identifying minority groups in each characteristic. Third, the optimal approach to inform clinical practice needs to be identified. In addition to concerns about comprehensibility of these candidate metrics, there may be specific clinical priorities; for example, some clinicians may not want to recommend a collection that includes many narratives about poor service experiences to their service users. Lastly, cultural adaptation of these metrics needs to be considered. For example, the Military veteran characteristic may hold more cultural importance in the United States than in many other countries [79]. In cultures such as Japan and South Korea, where age plays an important role [80], the Age characteristic may be more relevant. The next stages of research will include refinement of each metric with attention paid to minimizing the burden of calculation and developing interpretation guidance, the involvement of key stakeholders (ie, people living with mental health issues and mental health clinicians) in arbitrating between the candidate approaches, and real-world evaluation of the impact of more diverse and inclusive RRN collections.

## Appendix

Multimedia Appendix 1 Income level and Western, Educated, Individualized, Rich and Democratic (WEIRD)/non-WEIRD status of 20 countries.

Multimedia Appendix 2 Example calculations of the Simpson Diversity Index.

## Abbreviations

DMHI: digital mental health intervention
INCRESE: Inventory of Characteristics of Recovery Stories
NEON: Narrative Experiences Online
RRN: recorded recovery narrative
SDI: Simpson Diversity Index
SI: Simpson Index
WEIRD: Western, Educated, Individualized, Rich and Democratic
