# Supplementary material for: Assessing Diversity and Inclusivity is the Next Frontier in Mental Health Recovery Narrative Research and Practice
Source: JMIR Ment Health. 2023 Apr 17;10:e44601. doi: 10.2196/44601 (PMC10152384; doi:10.2196/44601)
Supplement: Multimedia Appendix 1 [file mental_v10i1e44601_app1.docx]

**Income level and WEIRD/non-WEIRD status of 20 countries**

| **Country** | **Income** | **WEIRD vs Non-WEIRD** |
| --- | --- | --- |
| Australia | High | WEIRD |
| Brazil | Upper Middle | Non-WEIRD |
| Cuba | Upper Middle | Non-WEIRD |
| Greece | High | Non-WEIRD |
| Guyana | Upper Middle | Non-WEIRD |
| Iran | Lower Middle | Non-WEIRD |
| Ireland | High | WEIRD |
| Italy | High | WEIRD |
| Japan | High | Non-WEIRD |
| Libya | Upper Middle | Non-WEIRD |
| Morocco | Lower Middle | Non-WEIRD |
| Netherlands | High | WEIRD |
| Norway | High | WEIRD |
| Palestine | Lower Middle | Non-WEIRD |
| Spain | High | WEIRD |
| Suriname | Upper Middle | Non-WEIRD |
| Tunisia | Lower Middle | Non-WEIRD |
| United Kingdom | High | WEIRD |
| United States | High | WEIRD |
| Yemen | Low | Non-WEIRD |

WEIRD = Western, Educated, Individualized, Rich and Democratic.
