# Supplementary material for: Assessing Diversity and Inclusivity is the Next Frontier in Mental Health Recovery Narrative Research and Practice
Source: JMIR Ment Health. 2023 Apr 17;10:e44601. doi: 10.2196/44601 (PMC10152384; doi:10.2196/44601)
Supplement: Multimedia Appendix 2 [file mental_v10i1e44601_app2.docx]

**Example** **calculations of the Simpson Diversity Index (SDI)**

RRN Collection X (20 narratives) comprises 10 narratives with female narrators, 10 narratives with male narrators, and 0 narratives with other gender narrators and 0 narratives in which the gender of the narrator was not identifiable. To calculate SDI for Collection X, the value for $N$ is 20 because there are 20 narratives in total (there is no narratives in which the gender of the narrator was not identifiable). The values for $n$ are as follows:

|  | ***n*** | ***n*(*n*-1)** |
| --- | --- | --- |
| Female | 10 | 90 |
| Male | 10 | 90 |
| Other | 0 | 0 |
| Not identifiable | 0 | 0 |
| **Total** | **20** | **180** |

SDI

= 1 - Σ*n*(*n*-1) / N(N-1)

= 1 - 10(10-1) + 10(10-1) + 0(0-1) + 0(0-1) / 20(20-1)

= 1 - 90 + 90 + 0 + 0 / 380

= 1 - 180 / 380

= 0.53

Collection Y (200 narratives) comprises narratives from 50 male narrators, 50 female narrators, 50 transgender narrators, 50 narratives with other gender narrators and 50 narratives in which the gender of the narrator was not identifiable. To calculate SDI for Collection Y, the value for $N$ is 200. The values for $n$ are as follows:

|  | ***n*** | ***n*(*n*-1)** |
| --- | --- | --- |
| Female | 50 | 2,450 |
| Male | 50 | 2,450 |
| Other | 50 | 2,450 |
| Not identifiable | 50 | 2,450 |
| **Total** | **200** | **9,800** |

SDI

= 1 - Σ*n*(*n*-1) / N(N-1)

= 1 - 50(50-1) + 50(50-1) + 50(50-1) + 50(50-1) / 200(200-1)

= 1 – 2,450 + 2,450 + 2,450 + 2,450 / 39,800

= 1 – 9,800 / 39,800

= 0.75

Therefore, the SDI score for Collection X is 0.53 and for Collection Y is 0.75, indicating Collection Y is more diverse than collection X.
